# Strain Tuning of Weyl Nodes in SrRuO_3_ Membranes

**DOI:** 10.1021/acs.nanolett.5c02997

**Published:** 2026-02-13

**Authors:** Patrick Blah, Stefano Gariglio, Edouard Lesne, Graham Kimbell, Dmytro Afanasiev, Jorrit Hortensius, Mattias Matthiesen, Dirk Groenendijk, Mafalda Monteiro, Mario Cuoco, Carmine Ortix, Andrea Caviglia

**Affiliations:** † Kavli Institute of Nanoscience, 2860 Delft University of Technology, P.O. Box 5046, 2600 GA Delft, The Netherlands; ‡ Department of Quantum Matter Physics, 27212 University of Geneva, 24 Quai E.-Ansermet, 1211 Geneva, Switzerland; § Institute for Molecules and Materials, Radboud University, P.O. Box 9010, 6500 GL Nijmegen, The Netherlands; ∥ CNR-SPIN, 84084 Fisciano, Italy; ⊥ Dipartimento di Fisica “E. R. Caianiello” Università degli Studi di Salerno, 84084 Fisciano, Italy

**Keywords:** oxide heterostructures, oxide membranes, perovskites, Weyl nodes, magnetotransport

## Abstract

Free-standing membranes are an exciting recent development in the field of complex oxides, allowing intrinsic material properties and phenomena to be probed in ways that would be difficult or otherwise inaccessible in epitaxially bound heterostructures. By employment of a water-soluble sacrificial layer of Sr_3_Al_2_O_6_, strain-free ultrathin SrRuO_3_ membranes have been fabricated that exhibit bulk lattice parameters and ferromagnetism at a Curie temperature of 150 K with the magnetic easy axis oriented 22° off the normal. The presence of sizable negative longitudinal magnetoresistance provides a direct signature of the decisive role played by Weyl Fermions in magnetotransport. In addition, a sign change between the strained films and free-standing SrRuO_3_ membranes of in-plane transversal magnetotransport indicates a strong electromechanical coupling, resulting in a change of the Fermi velocity of Weyl Fermions. Our measurements provide a first insight into the magnetoelectric properties of SrRuO_3_ membranes, highlighting the influence of the exfoliation process on structural, electronic, and magnetic degrees of freedom.

## Graphical abstract

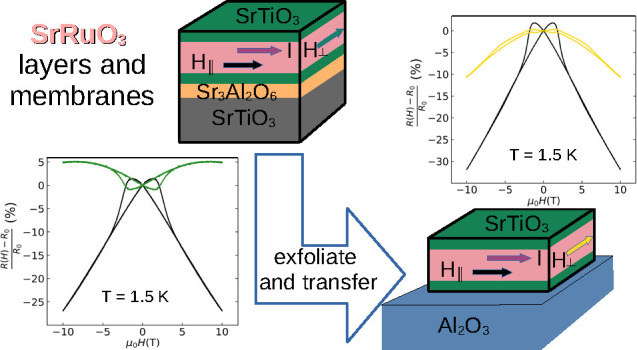

The strong coupling between the lattice and electronic properties is a characteristic feature of transition-metal oxides with perovskite structure. This is is due to the high susceptibility of the d orbitals to the crystal field of the oxygen octahedra. One of the many manifestations of this phenomenon is the magnetocrystalline anisotropy: in materials with strong spin–orbit coupling, the direction of the easy axis of the magnetization is linked to the orientation of the crystallographic axes. SrRuO_3_ (SRO), an orthorhombic (*Pbnm*) perovskite that hosts an itinerant ferromagnetic state below 160 K,^1^ illustrates this effect: its magnetization easy axis aligns along the *b* axis in single crystals^1^ and is highly sensitive to the strain state in thin films.^2^ Indeed, theory predicts that the magnetization can be completely quenched for large epitaxial strain.^3,4^ This also has profound consequences for the transport properties: the magnetic state couples strongly to the electrical conductance, as observed in the anomalous Hall effect (AHE).

Therefore, several studies have investigated the changes in magnetoelectric properties in SrRuO_3_ thin films by tuning their strain state through epitaxy.^5–8^

A key recent discovery has been the ability to add a sacrificial layer to epitaxially grown heterostructures^9^ to exfoliate the crystalline layers from their substrates and to transfer them onto any desired substrate using a dry stamping technique, resulting in thin, strain-free membranes that are no longer epitaxially bound to the substrate; this approach opens the way to induce new strain modulations^10^ or to couple materials with different symmetries^11^ and has been explored in SrRuO_3_ thin films.^12–15^ The aim of this work is to compare the structural, magnetic and magnetotransport properties of SrRuO_3_ before and after this exfoliation process. Analysis of SQUID measurements reveals that the strain release modifies the magnetic anisotropy, with the easy axis of the strain-free membranes rotated by 22° off the normal direction, where an easy axis parallel to the normal direction is typically observed for epitaxially bonded thin films. The AHE confirms the change in magnetization, showing a dramatic reduction in its amplitude. A negative longitudinal magnetoresistance (with collinear electric and magnetic fields) carries the signature of the Weyl nodes in SrRuO_3_. We also find a sign change of the in-plane transversal magnetoresistance, which is consistent with a reduction of the Fermi velocity of the Weyl nodes that occurs upon strain release.

SrRuO_3_ has an orthorhombic unit cell (*Pbnm*, *a* = 5.5670 Å, *b* = 5.5304 Å, and *c* = 7.8446 Å), which can be described as pseudocubic (pc) with a lattice parameter of 3.923 Å.^16^ Epitaxially grown on SrTiO_3_ (STO, cubic, *a* = 3.905 Å) substrates, SRO experiences a compressive strain of 0.47%.^17^ The Sr_3_Al_2_O_6_ (SAO, cubic, *a* = 15.844 Å ^18^) sacrificial layer has a good lattice match with STO (1.4% tensile strain), so it transfers the strain state set by the substrate in epitaxially bound heterostructures.

Panel a in Figure 1 illustrates the structure of the samples grown by pulsed-laser deposition: 15 unit cells (u.c.) of SAO were grown on a TiO_2_-terminated (001) STO substrate as sacrificial layer, followed by a layer of STO, 8 u.c. thick, and a layer of SRO 40 u.c. thick for sample A, 14 u.c. for sample B and 6 u.c. for sample C; this structure was capped with 8 u.c. of STO. A description of the exfoliation and transfer process, shown in Figure 1, is described in the Methods section.

**Figure 1:**
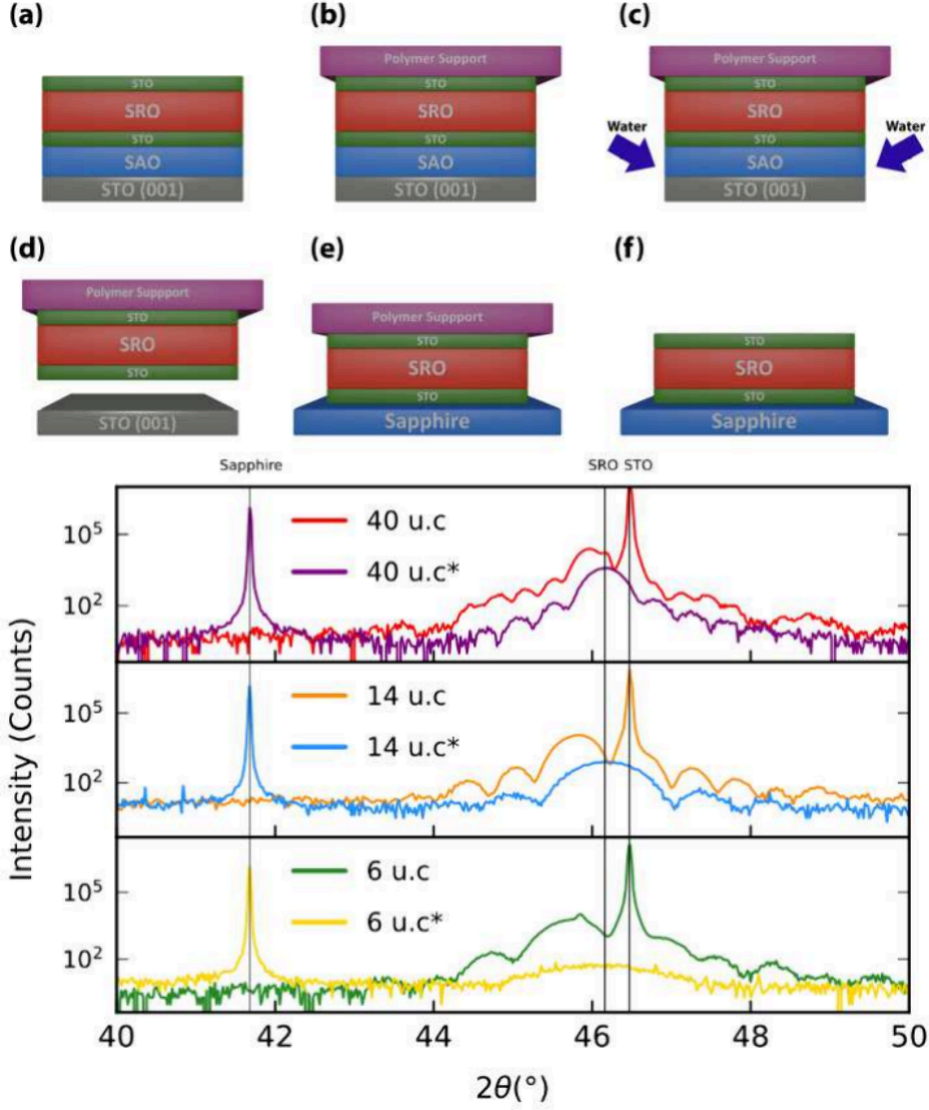
(Top) Illustration of the exfoliation and transfer technique. On top of the STO(001)/SAO/STO/SRO/STO heterostructure (a) is placed a PDMS stamp (b); the ensemble is immersed in deionized water for 24 h to dissolve the SAO layer (c). Once the SAO is dissolved, the substrate is separated from the epitaxial layers (d), which are stamped on a sapphire substrate (e). The PDMS stamp is removed, and the STO/SRO/STO membrane remains on the sapphire (f). (Bottom) XRD spectra of samples A (40 u.c.), B (14 u.c.), and C (6 u.c.) before and after exfoliation. The * indicates the exfoliated form of the sample. The vertical lines represent the bulk 2θ values of (0 0 0 6) sapphire, (0 0 2)_pc_ SRO, and (0 0 2) STO reflections. Note that the STO substrate is replaced by the sapphire after exfoliation.

Figure 1 shows the X-ray diffraction (XRD) spectra of the samples before and after exfoliation. A signature of the compressive strain is found in the position of the SRO (0 0 2)_pc_ 2θ peak, which shifts to a lower value due to the increase in the out-of-plane lattice parameter *c*_pc_ (sample A, 3.946 Å; sample B, 3.959 Å, sample C, 3.964 Å). After exfoliation, these peaks shift to the SRO bulk value, indicating that the SRO films are strain-free in their freestanding membrane form; the presence of finite size oscillations indicates that the membranes retain the high crystalline quality after the exfoliation and transfer process. A fit of these oscillations yields the thickness of the membranes in agreement with the deposition sequence (see the Supporting Information for data analysis).

The large spin–orbit coupling of the Ru 4d electrons determines the magnetocrystalline anisotropy observed in SRO: in its bulk form, the magnetization easy axis is oriented along the *b* axis. In thin films grown on (0 0 1) STO substrates, several studies have revealed that the epitaxial strain orients the magnetization along the film normal,^19,20^ as we have recently observed.^21^ Releasing the strain state leads to a reorientation of the easy axis: Figure 2 shows the temperature evolution of the remanence magnetization of sample B (after saturation) measured by SQUID magnetometry in the presence of a 5 mT magnetic field applied in-plane (*H*_∥_) and out-of-plane (*H*_⊥_). Additional figures showing a linear subtraction of the paramagnetic contribution can be found in the Supporting Information. From the values of the in-plane magnetic moment (*m*_∥_) and out-of-plane magnetic moment (*m*_⊥_) (Figure S5b), the easy axis was calculated to be oriented θ = 22 ± 2° off normal $\left(\theta =\arctan\;\frac{m_{∥}}{m_{⊥}}\right)$ .

**Figure 2:**
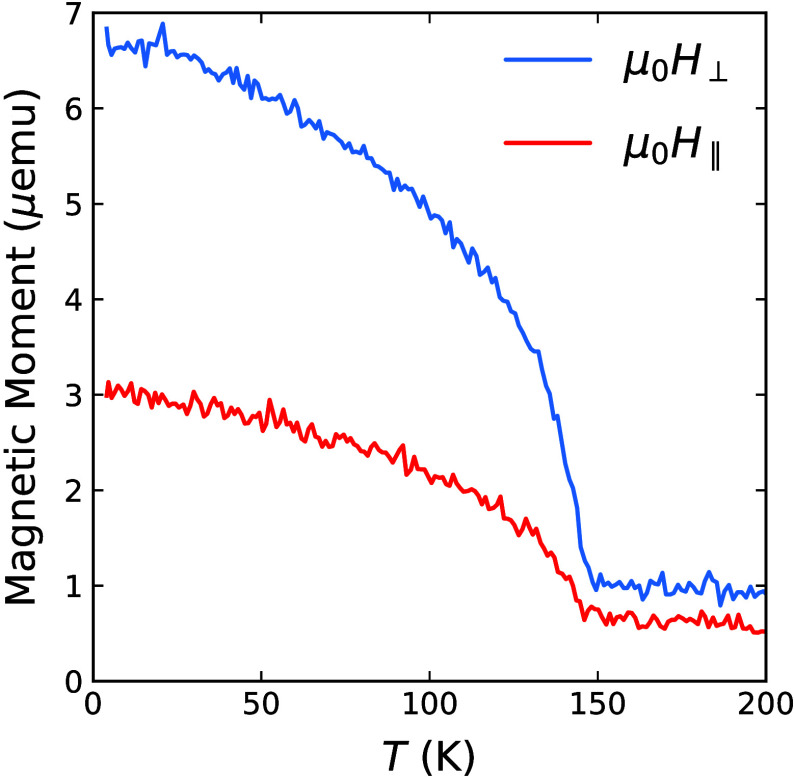
SQUID measurements of sample B performed with an external magnetic field applied in-plane and out-of-plane.

The change of the magnetic easy axis direction is a direct consequence of the coupling of the lattice with the d orbital electronic states: compressive strain causes the magnetization *M* to point more out-of-plane, while tensile strain causes it to point more in-plane.^5^ This is ascribed to compressive strain from the STO substrate increasing the tilting of the SRO octahedra^3^ resulting in larger orbital overlap of (d_*xz*_, d_*yz*_) orbitals with O p_*z*_ orbitals, where this overlap results in d_*xz*_, d_*yz*_ having a larger *z* component. Concordantly the SOI causes the magnetic moments to point more out-of-plane due to the increased *z* character of these orbitals that have preferential occupation.

The change in the direction of the magnetization *M* due to the release of the compressive strain after exfoliation is reflected in the electric transport. Figure 3 shows the longitudinal resistivity vs temperature of the three samples before and after exfoliation: as the temperature is reduced, all the curves display the characteristic kink indicative of the transition from the paramagnetic to the ferromagnetic state.^22^ The variation in resistivity values for different thicknesses may originate from an incorrect estimation of the geometrical factor of the devices. A comparison of the metallic behavior for sample B shown in Figure 3b reveals that the exfoliation and transfer process preserves the high conductivity of the sample over the whole temperature range, while the residual resistivity ratio attains a value of 3, for both the film and the membrane. The Curie temperature (*T*_C_), determined from the position of the kink in the resistivity, increases for samples A (137 K → 144 K) and B (145 K → 153 K; see the inset in Figure 3b), while it decreases for sample C (141 K → 136 K) and displays a broader kink (see the Supporting Information).

**Figure 3:**
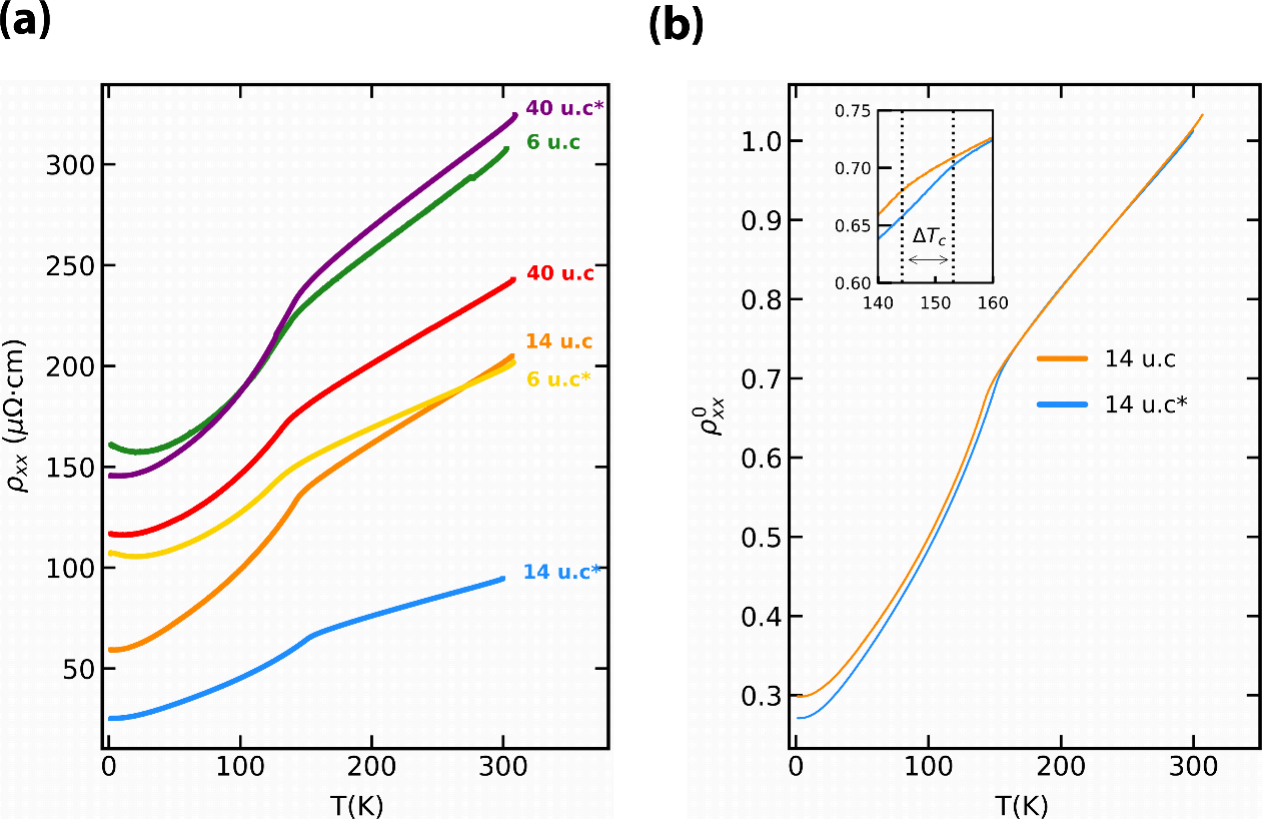
(a) Longitudinal resistivity vs temperature of samples A–C before and after exfoliation. The * indicates the exfoliated form of the sample. (b) Resistivity vs temperature for sample B before and after exfoliation. The data are normalized at room temperature. Inset: Zoom on the temperature range where the kink occurs. The dotted lines represent *T*_C_ before and after exfoliation, with Δ*T*_C_ representing the difference in *T*_C_. Δ*T*_C_ ≈ 7 K for sample B.

Striking evidence for the change in the magnetic state of the membranes with respect to the thin films appears also in the Hall effect data: the plots in Figure 4 illustrate the evolution of the transverse resistance for sample B as the temperature is varied across *T*_*C*_ (the data for samples A and C can be found in the Supporting Information). As commonly observed in SRO, the Hall effect shows an anomalous contribution as the temperature approaches the magnetic transition, with a concomitant change in sign: the shift in *T*_*C*_ observed in the longitudinal resistance is reproduced in the transverse component. A unique characteristic of the AHE in SRO is the fact that it changes sign at a certain temperature (*T*_switch_).^23^ This is attributed to the Berry curvature contribution to the intrinsic AHE, where the Fermi level crosses a band at a certain temperature which has a Berry curvature of the opposite sign.^24^ On the one hand, we observed an increase in *T*_switch_ after exfoliation. This could be attributed to the increased stability of the conduction band due to the increased overlap of the Ru 4d–O 2p orbitals,^25^ resulting in a larger thermal energy required to facilitate the crossing of itinerant electrons from one band to another. On the other hand, there is a large decrease of ρ_AHE_ after exfoliation. The amplitude of the anomalous contribution, which scales with the component of the magnetization perpendicular to the conducting plane, is drastically reduced for the membrane at low temperatures, in line with the change in the direction of magnetization observed in the SQUID measurements.

**Figure 4:**
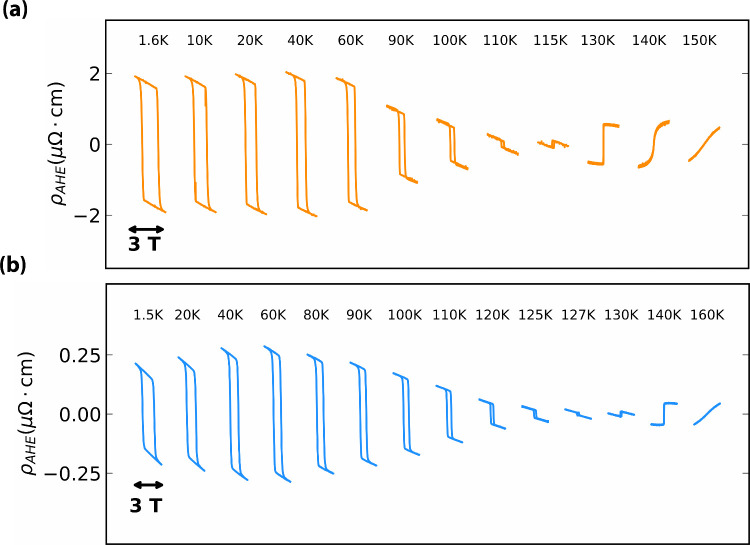
Anomalous Hall resistivity of sample B (a) before and (b) after exfoliation over a temperature range. The *x* axis represents the applied magnetic field strength, with each hysteresis loop being performed over 3 T (+1.5 to −1.5 T). The hysteresis loops are offset on the *x* axis for clarity.

To further probe the consequences of the change in the magnetic state of the membranes, we measured the magnetoresistance by applying the magnetic field in-plane along (μ_0_ *H*_∥_) and perpendicular to (μ_0_ *H*_⊥_) the electric current; a comparison of the measurements performed at 1.5 K in both geometries is displayed in Figure 5 for sample B (see the Supporting Information for the two other samples). From the parallel direction, the coercive field required for the magnetization reversal can be extracted: it decreases from 0.95 T before exfoliation to 0.695 T after exfoliation, confirming that the magnetic easy axis points more in-plane after the strain release. The presence of a negative linear longitudinal magnetoresistance at intermediate magnetic fields is consistent with an important role played by magnetic Weyl Fermions and their associated chiral anomaly. Strain relaxation does not quantitatively change the appearance of this negative longitudinal magnetoresistance, which is observed in all samples measured and is independent of sample thickness. The transversal longitudinal magnetoresistance displays instead a different behavior: the positive magnetoresistance at low fields observed in thin films turns negative for the membranes.

**Figure 5:**
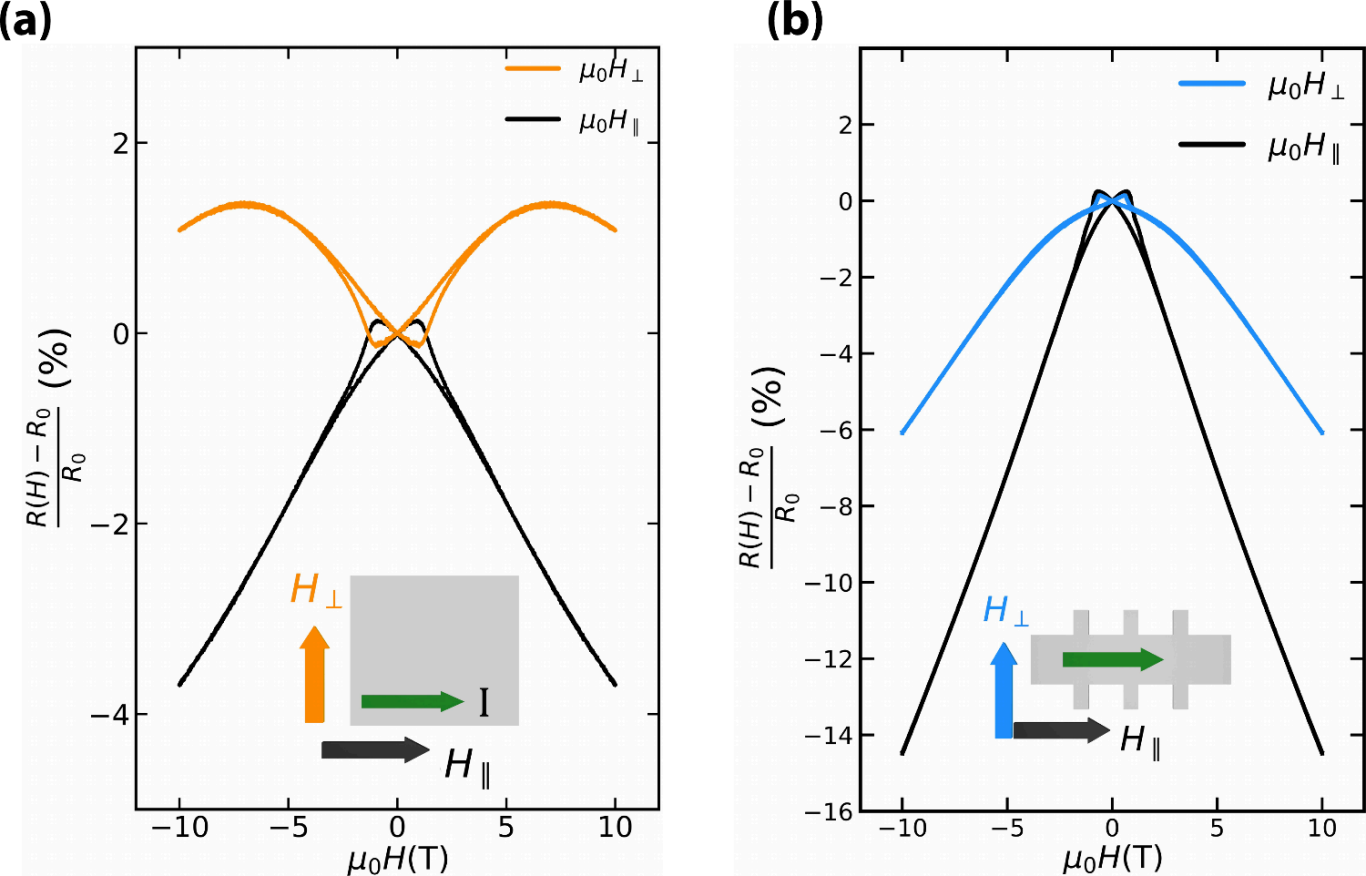
Magnetoresistance of sample B at a temperature of 1.5 K before (a) and after (b) exfoliation with the electric current parallel and perpendicular to an in-plane external magnetic field.

Figure 5 provides signatures that the magnetotransport of SrRuO_3_ membranes is mainly dictated by its Weyl Fermions. The negative longitudinal magnetoresistance observed before and after exfoliation is a well-known effect caused by the nontrivial Berry curvature associated with the Weyl cones. In addition, the magnetoresistance is strongly anisotropic, thus implying the concomitant presence of a planar Hall effect: a main fingerprint of the chiral anomaly.^26^ The existence of a finite transversal magnetoresistance and its change upon strain relaxation shows instead a strong coupling between electronic and lattice degrees of freedom.

A semiclassical Boltzmann transport theory predicts that the in-plane transversal magnetoresistance should be vanishing for ideal Lorentz-invariant Weyl cones.^26^ However, the presence of a tilt in the Weyl cones results in a finite in-plane transversal magnetoresistance^27^ even if they can be still classified as type I.^28^ Importantly, this transversal magnetoresistance can change from positive to negative if the tilt vector has a component along the driving electric field, with the sign change that is dictated by the ratio between the tilt parameter and the Fermi velocity. Therefore, the sign change can occur by decreasing the Fermi velocity at fixed tilt. This scenario is completely consistent with a release of compressive strain. As it occurs for instance in graphene, the effect of strain on Weyl cones is 2-fold:^29^ first, it affects the separation in momentum space between Weyl nodes of opposite chirality; second, and most importantly, it changes the Fermi velocity by an amount proportional to the strain strength. This provides an intuitive and simple explanation for the sign change in the transversal magnetoresistance observed in all membranes.

Ultrathin, metallic, strain-free SrRuO_3_ membranes of different thicknesses were created via water exfoliation of a sacrificial layer, Sr_3_Al_2_O_6_. Their structural parameters were investigated, showing that there is a release of compressive strain of the SrRuO_3_ films after exfoliation. Electronic transport measurements show an increase of the Curie temperature after exfoliation, highlighting the increased stability of the ferromagnetic phase due to the release of strain. Magnetotransport measurements display a large decrease in the anomalous Hall resistivity after exfoliation for each thickness, which can be attributed to the strain release causing the magnetic easy axis to point more in-plane. Additionally, these measurements provide signatures for strain-induced changes in the Fermi velocity of tilted Weyl nodes via exfoliation manifesting in a large reduction of the slope of the in-plane magnetoresistance, specifically when the applied magnetic field is perpendicular to the applied current. These flexible, itinerant ferromagnetic membranes provide a suitable platform to investigate geometric Berry phase driven phenomena such as the AHE, while highlighting the significant influence strain has on the material’s structural, electronic, and magnetic properties.

## Methods

SAO/STO/SRO/STO heterostructures were prepared via pulsed-laser deposition on a commercial TiO_2_-terminated SrTiO_3_ (001) substrate (provider: CryStec GmbH). The laser ablation was performed using a KrF excimer laser (Coherent LPXpro 305, λ = 248 nm) with a pulse frequency of 1 Hz. The growth conditions for each material layer can be found in the Supporting Information. The thickness was monitored *in situ* using reflection high energy electron diffraction (RHEED), and each sample was postannealed in an oxygen pressure of 300 mbar at 550 °C. The resulting heterostructures were measured via XRD. Following this, aluminum contacts were wire bonded to each sample in a van der Pauw geometry. Magnetotransport measurements were then performed using a He-flow cryostat equipped with a 10 T superconducting magnet. The samples were then exfoliated by attaching each to a PDMS stamp and being exposed to deionized water for 24 h. Each PDMS stamp was then stamped onto a commercial Al_2_O_3_ substrate (SurfaceNet). The XRD measurements were repeated for these SRO membranes on the sapphire substrates. Magnetometry measurements were made on the unpatterned membranes using a Quantum Design MPMS3 SQUID magnetometer in DC scanning mode. Holder and substrate signals were removed by measuring a bare sapphire substrate in the same holder under the same conditions and then subtracting and refitting the DC scans. The SRO flakes were electrically contacted by Pd (deposited by electron beam evaporation) using a combination of electron-beam lithography and lift-off technique. Prior to the Pd deposition, the insulating STO capping was etched *in situ* by Ar ion milling. The magnetotransport measurements in the He cryostat were then repeated for these flakes.

## Supplementary Material



## Notes

Experimental methods, deposition conditions, RHEED images, surface topography by atomic force microscopy, XRD patterns and their analysis, SQUID magnetometry, transport measurements, including resistivity, AHE, and magnetoresistance for an in-plane magnetic field (PDF)
