# Supplementary material for: Strain Tuning of Weyl Nodes in SrRuO3 Membranes
Source: Nano Lett. 2026 Feb 13;26(7):2356–61. doi: 10.1021/acs.nanolett.5c02997 (PMC12947686; doi:10.1021/acs.nanolett.5c02997)
Supplement: Supplementary file 1 [file nl5c02997_si_001.pdf]

Strain tuning of Weyl nodes in $SrRuO_3$ membranes
Supporting Information

Patrick Blah[1], Stefano Gariglio[2,*], Edouard Lesne[1], Graham Kimbell[2], Dmytro Afanasiev[3], Jorrit Hortensius[1], Mattias Matthiesen[1], Dirk Groenendijk[1], Mafalda Monteiro[1], Mario Cuoco[4], Carmine Ortix[5], Andrea Caviglia[2,*]

[1]*Kavli Institute of Nanoscience, Delft University of Technology, P.O. Box 5046, 2600 GA Delft, Netherlands.*
[2]*Department of Quantum Matter Physics, University of Geneva, 24 Quai E.-Ansermet, 1211 Geneva, Switzerland*
[3]*Institute for Molecules and Materials, Radboud University, P.O. Box 9010, 6500 GL Nijmegen, The Netherlands*
[4] *CNR-SPIN, 84084 Fisciano, Italy*
[5]*Dipartimento di Fisica "E. R. Caianiello" Università degli Studi di Salerno, 84084 Fisciano, Italy*
[*]*Corresponding authors: Stefano.Gariglio@unige.ch, Andrea.Caviglia@unige.ch*

(Dated: November 21, 2025)

I. GROWTH PARAMETERS

$Sr_3Al_2O_6/SrTiO_3/SrRuO_3/SrTiO_3$ heterostructures were prepared via pulsed laser deposition on a commercial TiO_2-terminated $SrTiO_3$ (001) substrate (provider: CryStec GmbH). The laser ablation was performed using a KrF excimer laser (Coherent LPXpro 305, $\lambda = 248$ nm) with a pulse frequency of 1 Hz.

**Layer**	**Temperature (°C)**	**Pressure (mbar)**	**Fluence* (J/cm^2)**	**No. Pulses**
SAO	750	1E-6	1.2	700
STO (1st)	550	1E-1	1	400
SRO sample A<br>SRO sample B<br>SRO sample C	550	1E-1	1	10000(40uc)<br>3500(14uc)<br>1555(6uc)
STO (2nd)	550	1E-1	1	400

* With a rectangular laser spot area of 2 cm^2

II. REFLECTION HIGH ENERGY ELECTRON DIFFRACTION PATTERNS

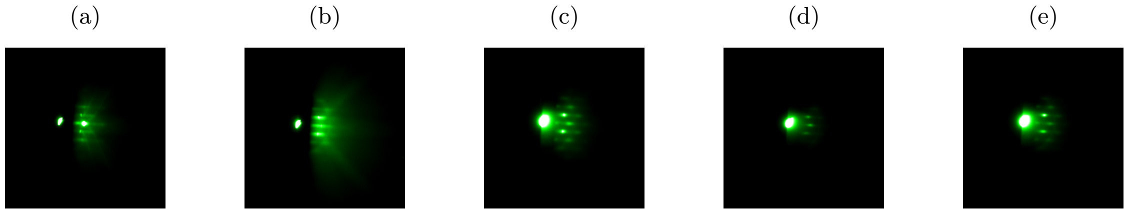

FIG. S1: Reflection high energy electron diffraction (RHEED) patterns recorded during the growth process of sample C: (a) (001) STO substrate (b) SAO layer (c) STO 1st layer (d) SRO layer (e) STO 2nd layer.

III. TOPOGRAPHY

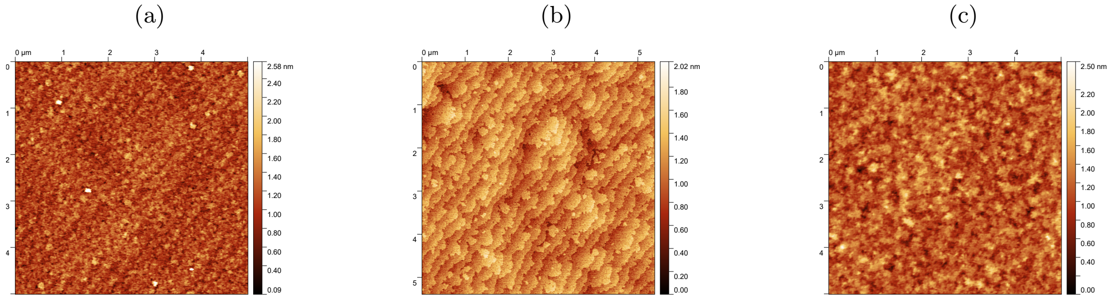

FIG. S2: Surface topographies by atomic force microscopy of (a) sample C (6 uc SRO), (b) sample B (14 uc SRO) and (c) sample A (40 uc SRO) before exfoliation.

IV. ANALYSIS OF X-RAY DIFFRACTION

sample	$2\theta(°)$	c_{out} (Å)	SRO thickness (uc)
SRO bulk	46.16	3.923	
sample A	45.96	3.946	40
Sample B	45.805	3.959	14
Sample C	45.74	3.964	6

TABLE I: 2θ values of $(0\,0\,2)_{pc}$ SRO reflection and corresponding pseudo-cubic out-of-plane lattice parameter (c_{out}) of samples with different SRO thickness measured with a Cu-$K_{\alpha 1}$ X-ray radiation.

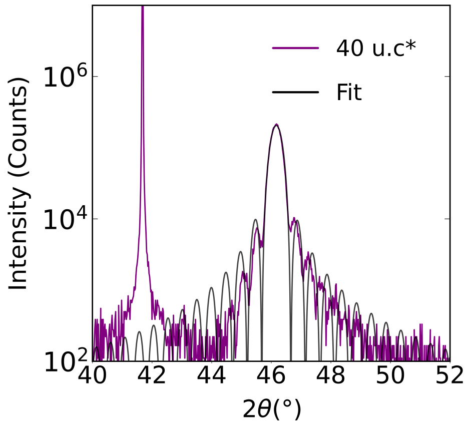

FIG. S3: Fitting of the $(0\,0\,2)_{pc}$ reflection of sample A after exfoliation: the fit (black line) yields a thickness of the membrane of 56 uc (8 uc of STO + 40 uc of SRO + 8 uc of STO).

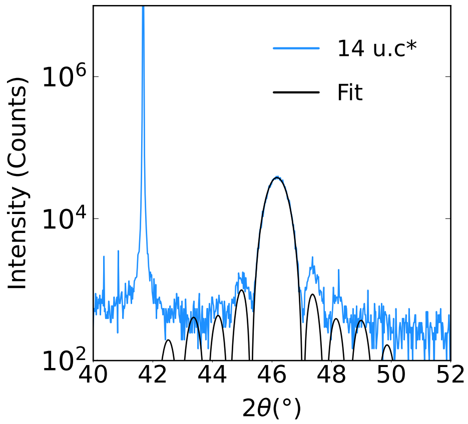

FIG. S4: Fitting of the $(0\,0\,2)_{pc}$ reflection of sample B after exfoliation: the fit (black line) yields a thickness of the membrane of 30 uc (8 uc of STO + 14 uc of SRO + 8 uc of STO).

V. SQUID

Figure 3 in the main text shows the magnetization of Sample B with an external magnetic field (5 mT) applied both in-plane and out-of-plane of the sample. It should be noted that this data was recorded via a remanence magnetization measurement. The measurement procedure was as follows: The sample was placed inside a plastic straw holder and inserted into the SQUID (MPMS3) cryostat at room temperature. The sample was then cooled from 300 K to 5 K. A large external magnetic field was applied (a few T) to saturate the magnetization of the SRO layer; this external magnetic field was then reduced to 5 mT. The sample was then warmed up to room temperature at a rate of 0.5 K per min while the magnetic moment of the sample was measured (known as $M(T)$ curves in the literature). The orientation of the sample was then changed, so that the external magnetic field from out-of-plane pointed in-plane to the sample, and the measurement procedure was performed again. An identical measurement procedure was performed on a clean sapphire substrate for control purposes. There were numerous reasons for using a 5 mT external magnetic field for the $M(T)$ measurements, namely: There is a large paramagnetic signal from the sample holder and substrate while applying large external magnetic fields, meanwhile the signal from the magnetic moment of the SRO layer is very small. Additionally there is always an unwanted negative residual field due to the trapped flux in the superconducting magnet of the SQUID (roughly 1 mT for MPMS3 instruments), hence an external magnetic field of 5 mT was applied to overcome this. The magnet was reset at room temperature after each measurement and the measurement procedure was identical for each measurement, hence the size and variability of the negative residual was greatly minimised.

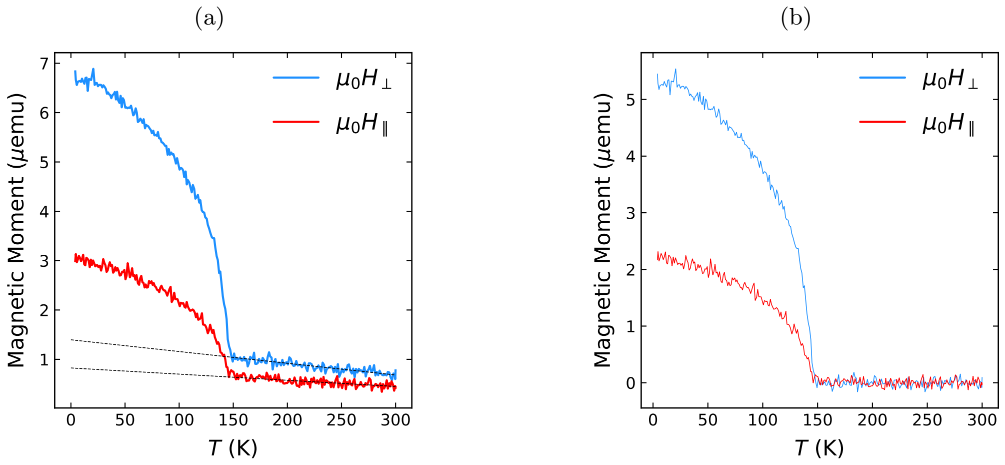

FIG. S5: (a) SQUID measurements of the remanence magnetization at 5 mT, where the dotted line represents the linear paramagnetic contribution from the sample holder. It should be noted that an additional paramagnetic contribution from the sapphire substrate was measured via a control experiment and subtracted from the data to produce this plot. (b) The remanence magnetization with the linear paramagnetic contribution subtracted. The estimation of the easy axis direction was performed from this plot.

VI. LONGITUDINAL RESITIVITIES

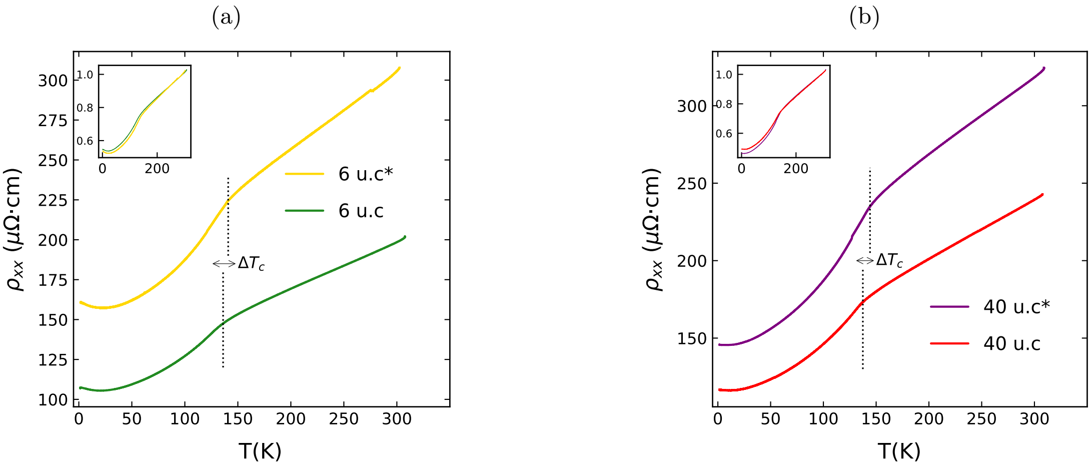

FIG. S6: Resistivity vs temperature before and after (*) exfoliation for sample A (a) and sample C (b). The vertical lines indicate the value of T_C . The data normalised at room temperature is shown in the inset.

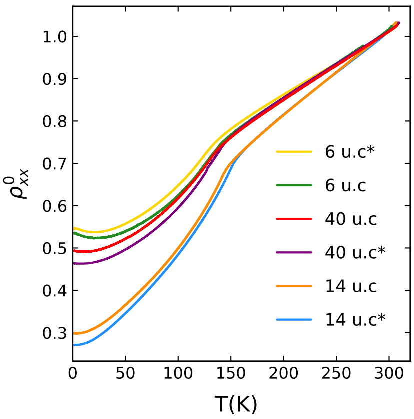

FIG. S7: Temperature evolution of resitivities normalised with respect to their room temperature values.

VII. ANOMALOUS HALL EFFECT

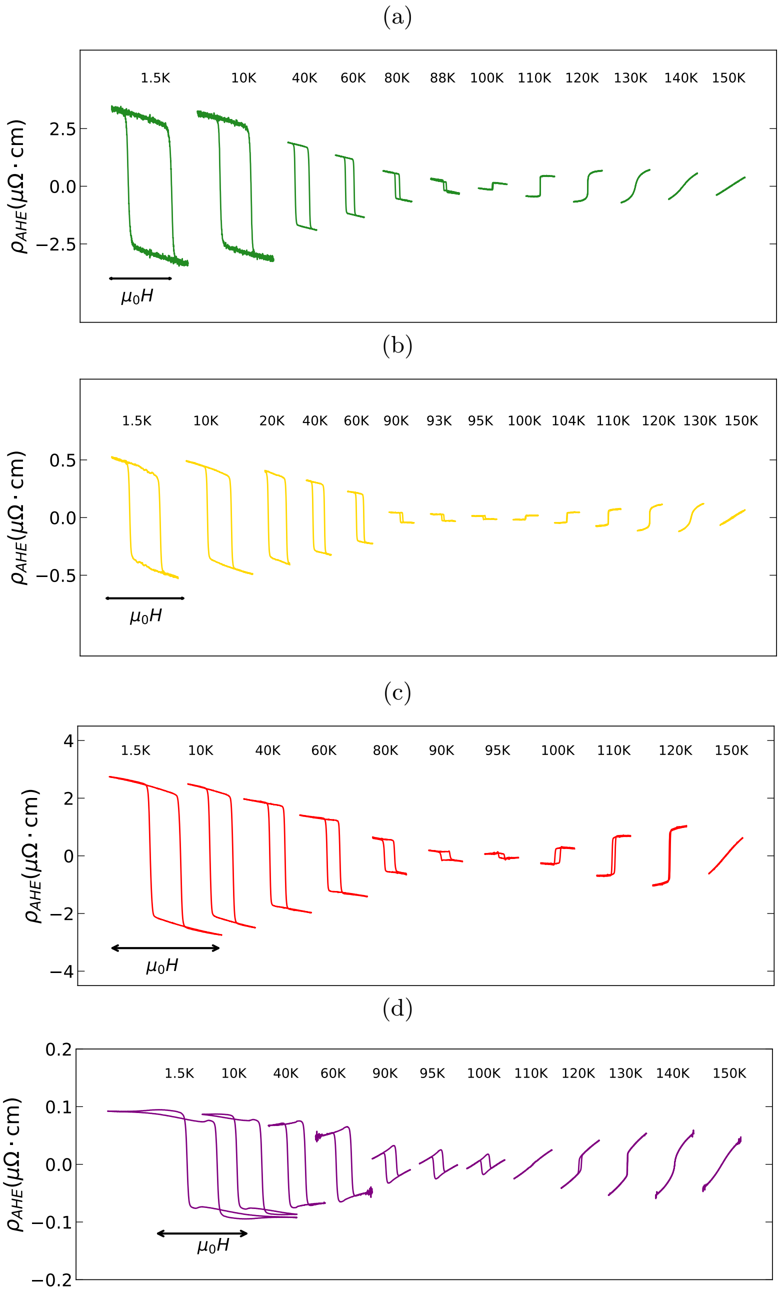

FIG. S8: Anomalous Hall resistivity before and after exfoliation for sample C (a)-(b) and sample A (e)-(f) plotted as a function of magnetic field for different temperatures.

The size of the magnetic field sweeps are different for certain loops shown in Figure S8. In more detail:

Sample	Temperature (inclusive) (K)	Total Field Sweep (T)
A	1.5-10	4
A	40-150	3
A*	1.5	20
A*	10	8
A*	40-60	6
A*	90-150	4
C	1.5-10	8
C	40-150	3
C*	1.5-10	8
C*	40-150	3

TABLE II: Amplitudes of the magnetic field sweeps applied at the different temperatures to the two samples for the anomalous Hall resistivity measurements. The * represents the exfoliated version of the sample.

VIII. MAGNETORESISTANCE

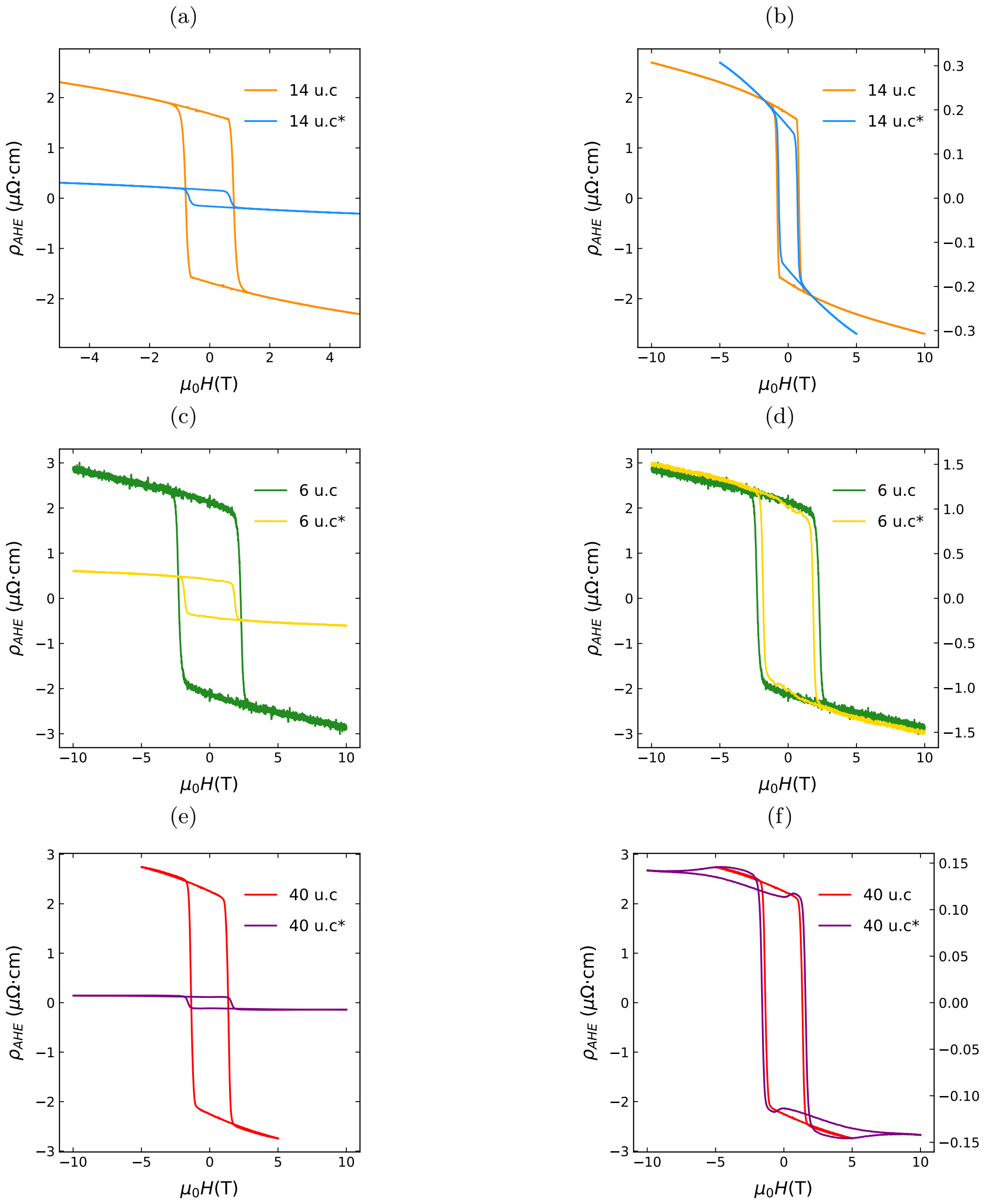

FIG. S9: Anomalous Hall effect of samples B (a), C (c), and A (e) at 1.5 K before and after exfoliation plotted on the same resistance axis. The plots on the right (b),(d),(f) use separate resistivity axes for the film and the membrane, revealing that the coercive field is not affect by the release of the epitaxial strain, only the magnitude of the Anomalous Hall resistance.

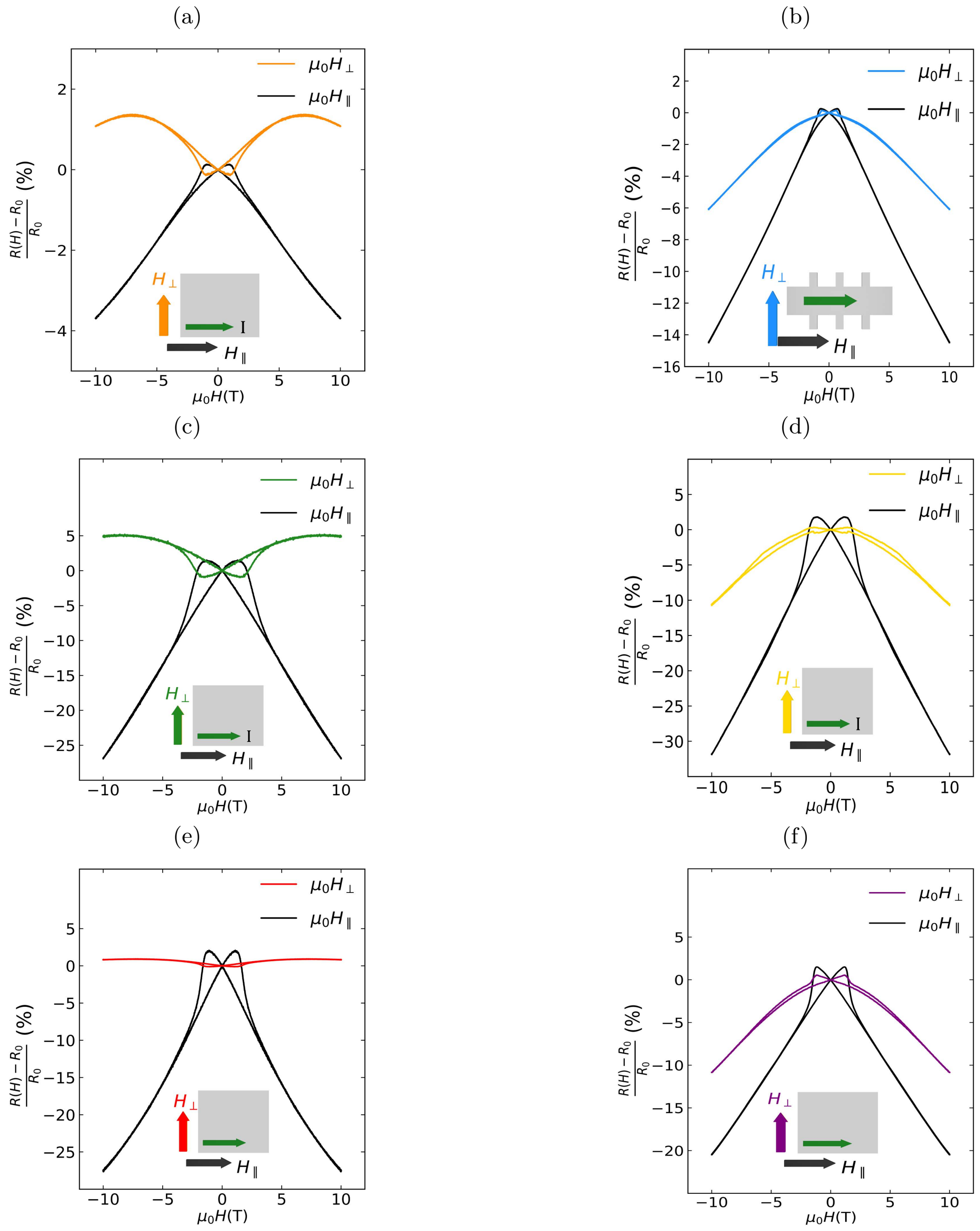

FIG. S10: Magnetoresistance of samples A (a), B (c) and C (e) before exfoliation, with electric current applied parallel and perpendicular to an in-plane external magnetic field. The corresponding magnetoresistance of sample A (b), B (d) and C (f) after exfoliation.
